# The Nitric Acid Test in the Examination of the Urine

**Published:** 1898-02

**Authors:** Charles E. Simon

**Affiliations:** Baltimore, Md.


### THE NITRIC ACID TEST IN THE EXAMINATION OF THE URINE.

BY CHARLES E. SIMON, M. D. BALTIMORE, MD.

In conformity with the instructions usually given in text-books on urinary analysis, the nitric acid test for albumin is generally employed in the following manner: A certain amount of urine is placed in a test-tube and nitric acid allowed to trickle down the sides of the tube, so as to form a distinct layer beneath the urine. In the presence of albumin, a cloudy ring will be seen to form at the zone of contact of the two fluids. If it is only desired to test a given urine for albumin, no objection can be made to this procedure. The amount of general information, however, which can thus be obtained is rather limited, and a great deal more may be learned from the specimen if a conical glass, of about two-ounce capacity, be used in place of the test-tube. This modification is quite generally accepted in the hospitals of France, and many of Germany, and undoubtedly deserves the attention of American physicians.

The glass is filled to about one-half of its capacity with the urine to be examined, when nitric acid is carefully added from the side, or through a pipette carried to the bottom of the vessel, so as to form a layer of about one-half to three-quarters of an inch in depth beneath the urine.

Under normal condition a brick-red to rose-colored band, referable to the presence of normal urinary pigments, is then observed at the zone of contact, while the urine itself remains perfectly clear. If albumin be present, however, a more or less pronounced cloudy ring will be seen immediately above and merging into the colored ring. Its extent and intensity vary with the quantity of albumin present, and it is possible with a little experience to form a fairly accurate idea of the total amount. To this end the depth of the albuminous ring should be accnrately measured and the amount of albumin determined separately with an Esbach albuminimeter. Bearing in mind the extent of the ring and the amount ascertained, it is possible, after a few experiments, to make an off-hand estimation from the qualitative examination alone. The same amounts of the reagent and of the urine, should, of course, always be employed, and it is convenient to mark the conical glasses accordingly. When it is desired to gain an insight into the amount of albumin eliminated in the twenty-four hours of the day, all the urine voided during this time should be carefully collected and a specimen taken from this collected amount for examination. Decomposition may be guarded against by placing in the receptacle about one tablespoonful of chloroform.

The cloud at the zone of contact may be due to serum albumin, serum globulin, albumoses or a mixture of these bodies. As serum globulin is always present when serum albumin is found, as a pure globulinuria has thus far not been observed, its significance is the same as that of serum albumin. If we wish to aseertain whether or not the precipitate contains albumoses, a small amount is removed with a pipette and heated over a spirit lamp or a Bunsen burner. Should albumoses only be present the cloudiness disappears, and the liquid in the presence of nitric acid turns a deep yellow color. Upon cooling, however, the precipitate reappears. In the presence of a mixture of serum albumin, serum globulin and albumoses, only a partial solution occurs, and the yellow color is not so marked.

A pure albumosuria is of special interest in so far as its existence should lead the physician to anticipate the appearance of serum albumin. Albumosuria may also alternate with true albuminuria.

A very important feature of this test, furthermore, is the fact that it furnishes an insight into the amount of uric acid eliminated. In order to obtain results of value, however, it is necessary always to work with specimens of urine taken from the collected amount of twenty-four hours. If uric acid be present in excess a distinct wafer-like band, resembling albumin in its general appearance, will be observed in clear urine *above* the zone of contact of the nitric acid and the urine. Should albumin be present at the same time it will be noticed that this band is separated from the albuminous ring by an intermediary zone of perfectly clear urine. If this point be remembered confusion will never arise, and it will not be necessary to study the effect of heat upon the individual precipitates in order to ascertain their true nature. If the uric acid ring does not appear after from five to ten minutes,it may be assumed that the substance is not present in increased amount, and that the quantity in all probability is even less than normal. As a general rule the band appears almost at once after the addition of the nitric acid, if an excessive elimination of uric acid has taken place; and from the rapidity with which it appears and the depth of the ring, an idea may be formed of the amount present, if the method has been carefully compared with one of the usual quantitative methods.

Occasionally, though rarely in the writer's experience, amounts of uric acid are encountered in the urine which are truly enormous, and almost immediately after the addition of the nitric acid a band of uric acid appears, which almost fills the entire bulk of urine and even extends to the nitric acid. Upon careful examination, however, it may be seen that the extension of the precipitate takes place from above downward, and not from below upward, as in the case of albumin. Should both be present in very large amounts at the same time, the decision, whether we are dealing with uric acid, or albumin, or both, may at first appear extremely difficult. The nitric acid should then be added through a pipette, and not allowed to flow along the walls of the vessel. Two bands can then always be made out for a few seconds at least, the one at the zone of contact, the other separated from this by an intermediary zone of practically clear urine.

Closely packed cotton stoppers impede its penetration. Wet covers or dressings almost entirely retard germicidal penetration. A number of authors have asserted that the agent exercises no deleterious action on the higher organisms. In the first experiment conducted by Dr. Harrington, of two rabbits left in the room where the gas was generated, one was found dead at the conclusion of the experiment and the other was found dead thirty-six hours later. The post-mortem findings in both cases showed great hyperemia, increased moisture of the respiratory passages and congestion of the lungs. This would indicate that the claims on this point are not well founded.

In this same connection, Drs. Reik and Watson have reported in the *Johns Hopkins Medical Bulletin* some experiments for sterilizing instruments with this agent. Knives which had been used in dissecting were washed in water and placed in a 1-2,000 formaldehyde solution. It was found that in thirty-five minutes they were completely sterilized. Where gas is generated in a small chamber considerable experimentation was necessary to learn the percentage of gas necessary to sterilize instruments within a reasonable length of time, such as ten or fifteen minutes. In a chamber containing a cubic foot of air it was found that gas generated from five to three grains of paraform (polymerized formaldehyde) was sufficient to produce practical sterilization in fifteen minutes. Of course a great deal of interest attaches to the question whether or not the agent has any deleterious effect upon instruments. As yet we can only say that no such action has been noted, either in the way of rusting or dulling.

It would seem, then, that this agent promises to be quite useful in this way. Used in a chamber of one foot cubic space, the expense of such disinfection, including the cost of paraform and alcohol, will not exceed one cent. For the disinfection of small instruments, such as those used by ophthalmologists, otologists, laryngologists and dentists, it is by far the most convenient and speedy method.

To this it might be added, also, that the experiments indicate that not much reliance can be placed on the method of evaporating watery solutions of formalin in open vessels, the moisture thrown into the air and deposited on the materials to be disinfected, impeding the germicidal action of the gas.—*Kansas City Medical Index.*

